# Glenoid concavity has a higher impact on shoulder stability than the size of a bony defect

**DOI:** 10.1007/s00167-021-06562-3

**Published:** 2021-04-11

**Authors:** Jens Wermers, Benedikt Schliemann, Michael J. Raschke, Philipp A. Michel, Lukas F. Heilmann, Felix Dyrna, Julia Sußiek, Andre Frank, J. Christoph Katthagen

**Affiliations:** Department of Trauma, Hand and Reconstructive Surgery, University Hospital Münster, Waldeyerstraße 1, 48149 Münster, Germany

**Keywords:** Concavity compression, Glenoid bone loss, Shoulder instability, Stability ratio, Bony shoulder stability ratio, Glenoid concavity

## Abstract

**Purpose:**

Surgical treatment of shoulder instability caused by anterior glenoid bone loss is based on a critical threshold of the defect size. Recent studies indicate that the glenoid concavity is essential for glenohumeral stability. However, biomechanical proof of this principle is lacking. The aim of this study was to evaluate whether glenoid concavity allows a more precise assessment of glenohumeral stability than the defect size alone.

**Methods:**

The stability ratio (SR) is a biomechanical estimate of glenohumeral stability. It is defined as the maximum dislocating force the joint can resist related to a medial compression force. This ratio was determined for 17 human cadaveric glenoids in a robotic test setup depending on osteochondral concavity and anterior defect size. Bony defects were created gradually, and a 3D measuring arm was used for morphometric measurements. The influence of defect size and concavity on the SR was examined using linear models. In addition, the morphometrical-based bony shoulder stability ratio (BSSR) was evaluated to prove its suitability for estimation of glenohumeral stability independent of defect size.

**Results:**

Glenoid concavity is a significant predictor for the SR, while the defect size provides minor informative value. The linear model featured a high goodness of fit with a determination coefficient of *R*^2^ = 0.98, indicating that 98% of the SR is predictable by concavity and defect size. The low mean squared error (MSE) of 4.2% proved a precise estimation of the SR. Defect size as an exclusive predictor in the linear model reduced *R*^2^ to 0.9 and increased the MSE to 25.7%. Furthermore, the loss of SR with increasing defect size was shown to be significantly dependent on the initial concavity. The BSSR as a single predictor for glenohumeral stability led to highest precision with MSE = 3.4%.

**Conclusion:**

Glenoid concavity is a crucial factor for the SR. Independent of the defect size, the computable BSSR is a precise biomechanical estimate of the measured SR. The inclusion of glenoid concavity has the potential to influence clinical decision-making for an improved and personalised treatment of glenohumeral instability with anterior glenoid bone loss.

**Supplementary Information:**

The online version contains supplementary material available at 10.1007/s00167-021-06562-3.

## Introduction

The mechanism of concavity compression is known to be a key factor for glenohumeral stability in the mid-range of motion due to a laxity of the capsule and ligaments [7, 13, 15]. The humeral head is actively pressed into the glenoid cavity by the rotator cuff with a compression force of up to 81% of the body weight during an abduction movement [1]. The more intensely this medial compression force is exerted, the more force is required to trigger an anterior dislocation, as the humeral head must move anterolaterally across the glenoid rim. The ratio of the maximum dislocating force the joint can resist related to the medial compression force is defined as the stability ratio (SR). The SR is given as a percentage and is frequently used in numerous biomechanical studies focusing on glenohumeral instability [5, 7, 8, 13, 15, 29].

The SR may be affected by prevalent injuries such as a Bankart lesion, a fracture of the anterior glenoid rim, or by chronic bone loss [7, 8, 13, 22, 29]. Small defects can be treated well with an arthroscopic Bankart repair, whereas significant bone defects lead to a recurrence rate of up to 67% when treated with a soft tissue reconstruction alone [3]. A bone loss of 20–25% relative to the glenoid extent of the short axis, hereinafter referred to as defect width, is a widely accepted threshold at which bony reconstruction is preferred over soft tissue Bankart repair [6, 8, 16, 29]. However, current studies and clinical outcomes recommend lower thresholds to indicate subcritical or minor bone loss [11, 24, 25, 30]. In addition, Giacomo et al. and Lacheta et al. recently stated that factors other than the degree of bone loss should be considered to tailor the treatment more closely to the patient [4, 12].

The defect size as a single decisive criterion for the surgical treatment was recently challenged by Moroder et al. [18, 19]. Instead of adapting the critical threshold, they postulated the glenoid concavity as another important parameter for stability in their finite-element method (FEM)-based study. The authors proved that constitutional differences in glenoid shape led to significant biomechanical implications. In addition, they derived the bony shoulder stability ratio (BSSR), a mathematical approximation of the SR independent of defect size [19–21]. In principle, the BSSR is based on measurements of the bony structure and can be computed from CT data by measuring the glenoid depth and the sphere radius of the humeral head. Therefore, the BSSR could be established clinically as a supplement to the defect size measurement. Moroder et al. thus introduced a theoretical background for a new concept of assessing glenohumeral stability, which to date has not been supported by biomechanical research.

This biomechanical study focused on the stabilising role of glenoid concavity depending on the defect size of anterior glenoid bone loss. The objective was to prove if glenoid concavity might be a suitable parameter for a more customised surgical treatment in the future. It was hypothesised that the SR depends mainly on glenoid concavity and that the initial concavity affects the loss of stability caused by bony defects. Results of this study could thus optimise the treatment of shoulder instability, which is currently based on bony defect size, toward a more personalised approach based on concavity.

## Materials and methods

### Specimen preparation

A total of 22 human cadaveric scapulae were obtained from the University of Lübeck, Germany with institutional review board approval (IRB No. 2014-421-f-N, University of Münster, Germany). All donors provided written consent to use their bodies for scientific and/or educational purposes. The specimens were thawed overnight, and all soft tissue was dissected to focus on the effects of osteochondral integrity on glenohumeral stability. The acromion and the coracoid process (CP) were removed from the scapulae to ensure a standardised movement of the humeral head. The cartilage was moistened with saline during the entire testing procedure.

**Fig. 1:**
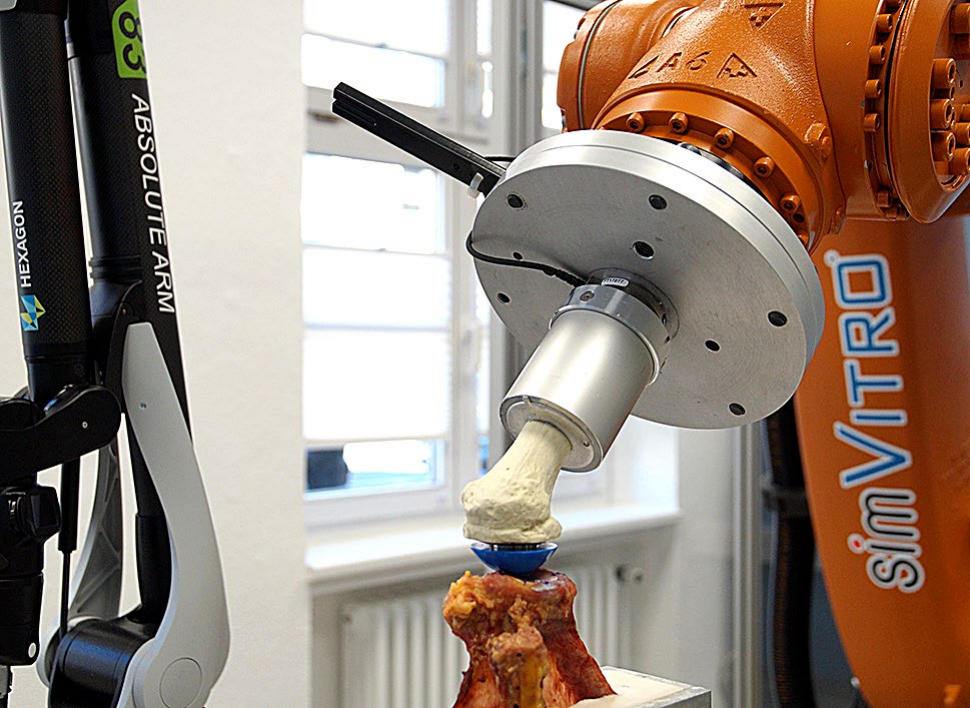
Human specimen in robotic test setup; experimental setup consisting of 3D measuring arm, industrial robot, force torque sensor, artificial bone with stemless shoulder implant and human cadaveric scapula. The humeral head is shifted in the 3 o’clock direction to perform an anterior dislocation

Five glenoids suffered from macroscopically visible osteoarthritis and were excluded from testing. The remaining *n* = 17 specimens [11 right, 6 left, 10 females, 7 males, age 79.06 ± 8.68 (62–93) years] were embedded with polyurethane casting resin (RenCast PU, Gößl + Pfaff GmbH, Karlskron/Brautlach, Germany) in a custom-made frame. The casting mould was positioned in the test setup with the glenoid plane aligned horizontally with the floor (Fig. 1 and additional file 1). To focus on osteochondral effects of the glenoids, and to minimise influences of cartilage or bone defects at the human cadaveric humeri, artificial proximal humeri (1028/1028-20, Sawbones, Malmö, Sweden) were potted and equipped with stemless shoulder implants and trial heads (Eclipse, Arthrex GmbH, Munich, Germany). Size and radius of the implants were chosen individually for each glenoid according to a best-fit approach. Two surgeons selected the trial heads according to their clinical experience, starting with the smallest head and increasing the size until the glenoid cavity was completely covered. If both surgeons yielded different sizes, the smaller size was chosen to ensure that the trial head was always in contact with the deepest point of the glenoid cavity.

### Digitization and test setup

The implanted artificial humeri were mounted together with a force torque sensor (Mini45, ATI Industrial Automation, Apex, USA) onto a six-axis industrial robot (KR 60-3, KUKA, Augsburg, Germany) (Fig. 1). The accuracy of position repeatability was 60 µm, and the force measurement error was less than 0.25 N. Robot control was performed with simVITRO (Cleveland Clinic BioRobotics Lab, Ohio, USA), a software for robotic joint testing.

**Fig. 2:**
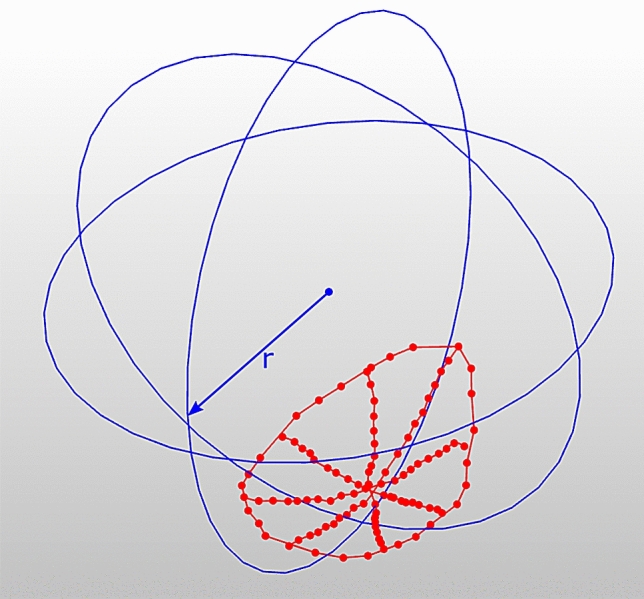
Morphometric measurements of the intact glenoid; the 3D measurements were used to determine features of the glenoid (red) such as length and width as well as the sphere radius of a best-fit humeral head (blue), hereinafter referred to as concavity radius (*r*)

Anatomical landmarks of each glenoid and humerus were digitised using a 3D measuring arm (Absolute Arm 8320-7, Hexagon Metrology GmbH, Wetzlar, Germany) with a measurement error of less than 50 µm. To ensure glenoid-specific translations, a joint coordinate system was defined for each glenoid. The superoinferior and anteroposterior axes of this joint coordinate system were aligned with the long and short axes of the glenoid, respectively. By performing the robot movement in these axes, the effects of a physiological version could be minimised as well as effects of a version due to minimal potting inaccuracies. The 3D measuring arm was further used to capture the articular surface of the glenoid. For this, a total of more than 100 surface points were measured (Fig. 2 and additional file 1).

### Biomechanical experiments

**Fig. 3:**
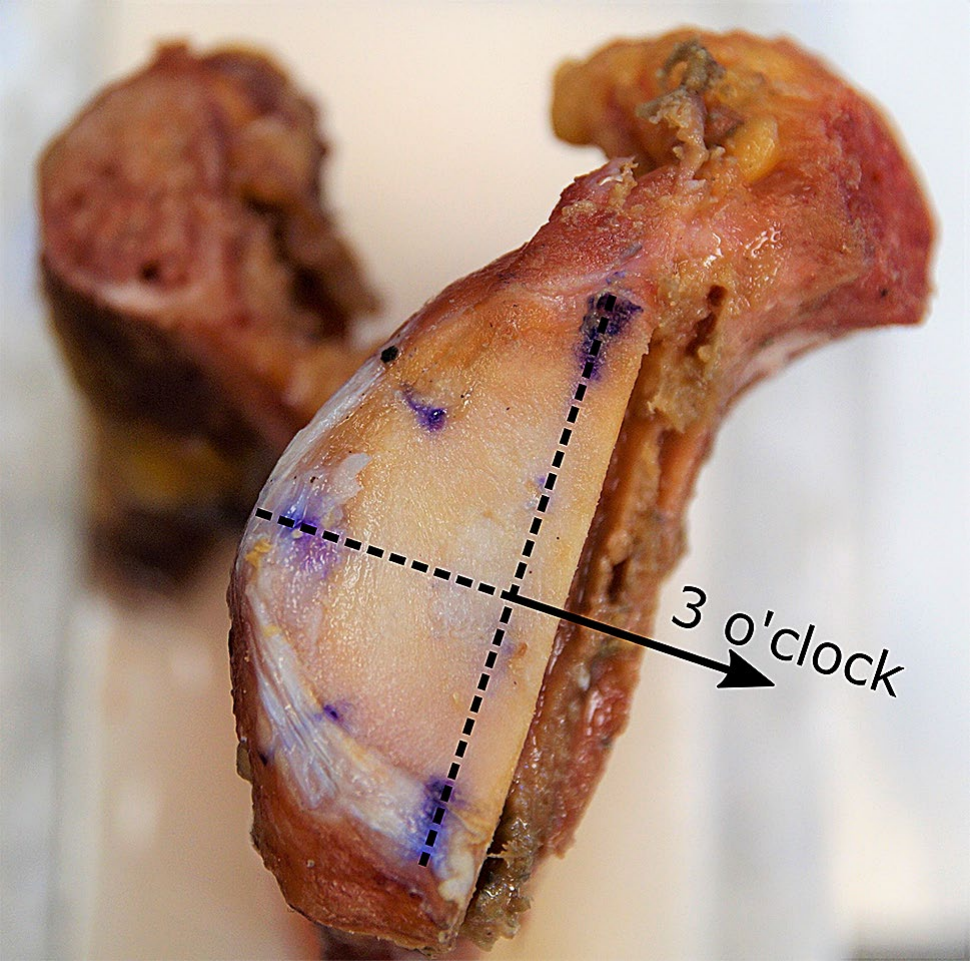
Right human cadaveric glenoid with artificial bone defect; the defect line is aligned parallel to the long axis of the glenoid. Anterior dislocations were performed in the 3 o’clock direction

**Fig. 4:**
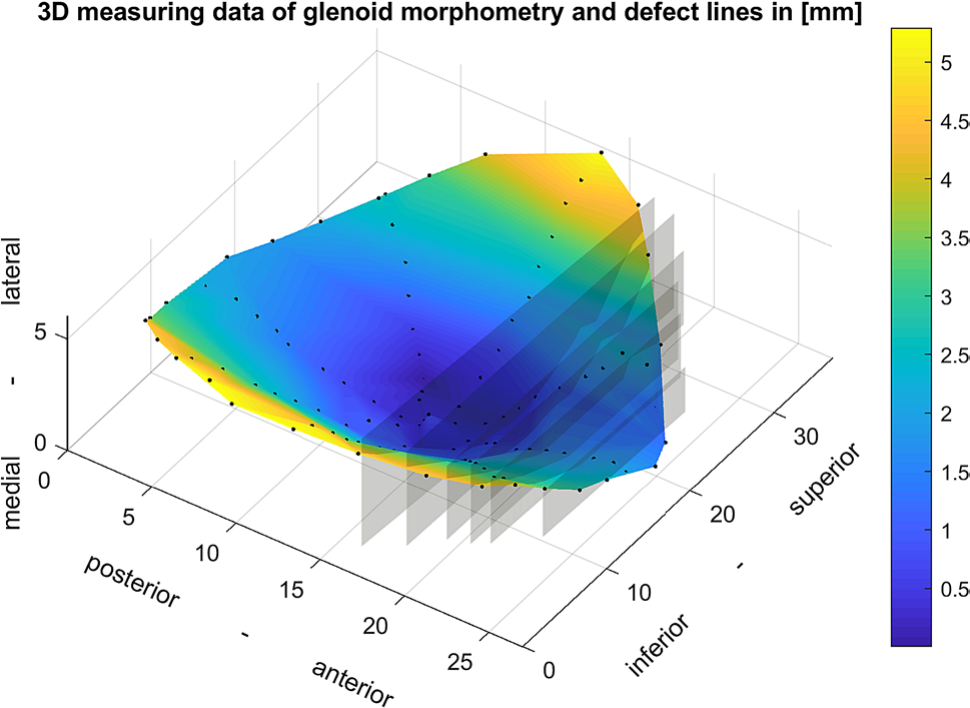
Processed 3D measuring data of intact glenoid and true artificial defect lines; the colour bar illustrates the mediolateral glenoid extent equivalent to the glenoid depth

Testing was performed in glenohumeral abduction of 56° (corresponding to 90° humerothoracic elevation) and neutral internal rotation [17]. This elevation was chosen to simulate a load-and-shift test, in which soft tissues are rather lax, and stability is mainly provided by the articular surface [8]. The humeral head was positioned manually inside the glenoid cavity. A medial aligned compression force of 50 N was exerted through the robot while all other forces were minimised. Thereby, a neutral starting position was achieved. The compression of 50 N, frequently used for the determination of the SR in the past, was continuously maintained to keep the humeral head uniformly compressed in the glenoid cavity [8, 13, 15, 22, 29, 31]. Anterior dislocations were performed by shifting the humeral head in the 3 o’clock direction relative to a right scapula (Fig. 3). Bony defects were created parallel to the long axis at the anterior glenoid rim by means of a hand-guided rotary tool (Multitool 4000, Dremel, Breda, Netherlands). This defect orientation is typically associated with osseous anterior defects [2, 23]. The defect size was increased in steps of 2 mm width (additional file 1). For each defect, dislocations were repeated, and the defect line was digitised for evaluation of the true defect size (Fig. 4). Defect creation was stopped when dislocation of the humerus occurred only by application of compression.

**Fig. 5:**
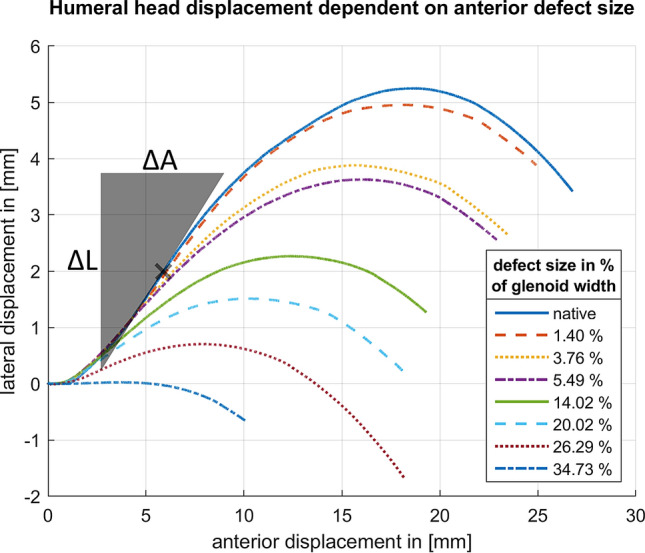
Displacement of the humeral head for a single specimen and different defect sizes; the maximum lateral displacement is used for estimation of glenoid depth (*d*). The concavity gradient is determined as the maximum ratio of lateral displacement (Δ*L*) related to anterior displacement (Δ*A*)

The following characteristics were evaluated for each defect step. The true defect size was determined as a percentage of the intact glenoid width to report the results independent of glenoid size. The SR was obtained as a percentage of the maximum anterior force required for dislocation divided by the constant compression force of 50 N. The robot movement was used to acquire the lateral and anterior displacement of the humeral head. This displacement is shown in Fig. 5 for a single specimen with different defect sizes. Furthermore, the bony shoulder stability ratio (BSSR) was computed with the following equation as derived by Moroder et al. [20]:

$$ {\text{BSSR}} = \frac{{\sqrt {1 - \left( {\frac{r - d}{r}} \right)^{2} } }}{{\frac{r - d}{r}}} $$

In this equation, (*r*) is the concavity radius, and (*d*) is the glenoid depth. The concavity radius (*r*) equals the sphere radius of a best-fit humeral head, which was determined from digitised surface points of the glenoids. The glenoid depth (*d*) equals the maximum lateral displacement of the robot. In addition, the curvature of the displacement was analysed as follows: the slope of a tangent line in a single point was calculated as the ratio of lateral displacement (Δ*L*) to anterior displacement (Δ*A*). This ratio was also denoted as the gradient in this single point. The maximum occurring gradient for a displacement curve was then determined as the maximum ratio of lateral displacement (Δ*L*) to anterior displacement (Δ*A*) for all data points. This value was determined for each defect step and defined as concavity gradient given as a percentage.

### Statistical analysis

A custom-made MATLAB-Script (R2019a, The MathWorks Inc., Massachusetts, USA) was developed for signal processing of all characteristics in each defect step. For all statistical evaluations, non-parametric linear mixed-effects models were deployed. The significance level was set to *p* < 0.05, and the resulting linear coefficients are presented as mean ± SE (95% CI).

A first linear model was established using the defect size and concavity gradient as predictors for the SR. The linear coefficients of both predictors were evaluated to compare their impact on the SR. The model determination coefficient (*R*^2^) and the mean squared error (MSE) were used as outcome measurements for the correlation. A second linear model was determined using only the defect size as a predictor for the SR to evaluate its expressiveness without usage of concavity information.

The loss of SR with increasing defect size was further analysed by classifying the specimens according to their initial concavity into three equal groups of low (< 25%), medium (25–35%) and high (> 35%) initial concavity gradients. For each group, a separate linear model with defect size as a single predictor for the SR was set up. The coefficients of these models were compared to prove the impact of initial concavity on the loss of SR caused by bone defects. Lastly, the correlation between the morphometric-based BSSR and the measured SR was analysed in a linear model to examine the suitability of the BSSR as a predictor in clinical daily routine.

For an estimated correlation coefficient of *r* = 0.7 based on biomechanical studies focusing on the SR [13, 15], a sample size of 17 is required to demonstrate a significant effect with a statistical power of 0.9 and a significance level of *p* < 0.05.

## Results

**Fig. 6:**
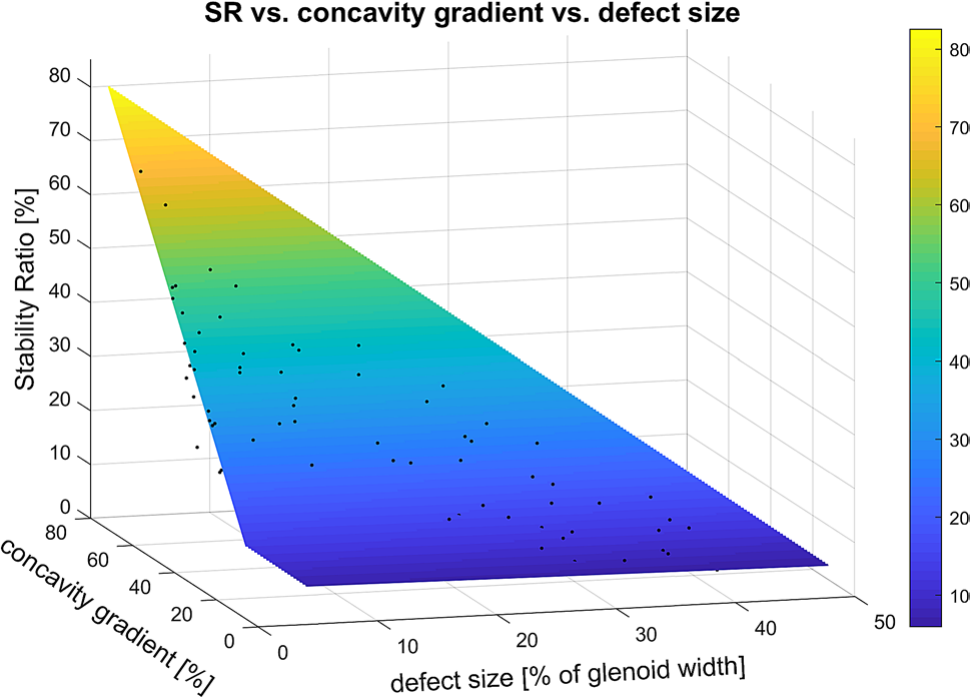
Linear model with concavity gradient and defect size as predictors for the SR; the SR is mainly dependent on the concavity gradient whereas the defect size has a minor, indirect influence

The characteristics of all *n* = 17 specimens are summarised in Table 1 for the intact state. The first linear model with glenoid concavity and defect size as predictors for the SR is depicted in Fig. 6.

**Table 1:** Initial morphometric measurements and outcome parameters presented as mean ± standard deviation (range)

Morphometric measurements		Outcome parameters	
Glenoid length in mm	32.3 ± 3.6 (26.4–41.0)	Concavity gradient in %	30.8 ± 9.9 (17.5–56.0)
Glenoid width in mm	25.1 ± 3.0 (18.7–32.6)	Bony shoulder stability ratio (BSSR) in %	39.2 ± 12.1 (23.3–67.8)
Glenoid depth (d) in mm	2.2 ± 1.1 (0.7–5.3)	Stability ratio (SR) in %	39.6 ± 12.0 (24.1–70.4)
Concavity radius (r) in mm	31.3 ± 2.9 (25.4–35.8)

The linear coefficient for the effect of concavity gradient averaged 1.1 ± 0.1 (95% CI [1.0, 1.1]), meaning that the SR increases by 1.1% with each percentage of concavity gradient. This coefficient differed significantly from zero with *p* < 0.001. The defect size had only a minor and indirect impact on the SR, as the linear coefficient of − 0.03 ± 0.04 (95% CI [− 0.1, 0.1]) was insignificantly different from zero with *p* = 0.53. The linear model featured a high goodness of fit with a determination coefficient of *R*^2^ = 0.98 corresponding to a high predictability of the SR of 98% by concavity gradient and defect size. The low MSE of 4.2% also indicates a high quality of estimation. In a second linear model, the defect size was applied as an exclusive predictor for the SR. This reduced *R*^2^ to 0.87 and increased the MSE to 25.7%, thus, degrading both outcome parameters. These results reveal a more precise estimation of the SR when using not only the defect size but also the concavity gradient as a predictor.

**Fig. 7:**
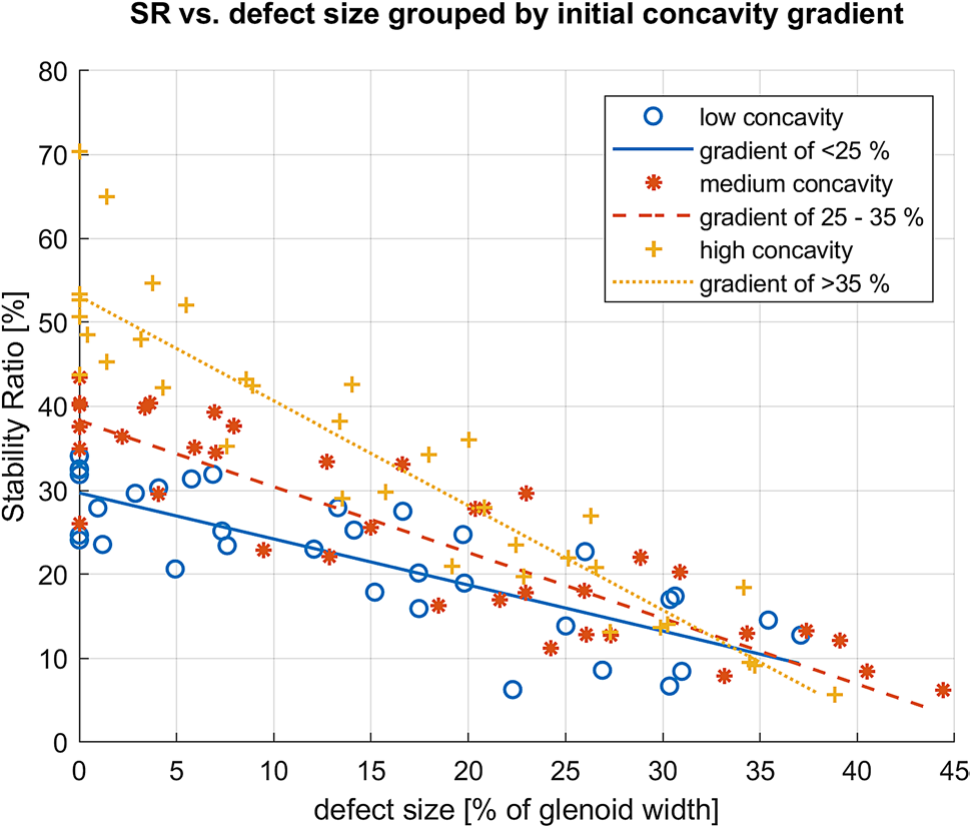
Linear models with defect size as predictor for the SR; the specimens were classified in three groups by their initial concavity gradient. The decline of the linear slopes corresponds to the loss of SR with increasing defect size

The separate linear models for each group of low (< 25%), medium (25–35%) and high (> 35%) initial concavity gradients incorporating the defect size as a single predictor for the SR are shown in Fig. 7. Statistics of the three linear models are listed in Table 2. The mean loss of SR due to increasing defect size differs significantly among the three groups of initial concavity gradients. The group with the highest initial concavity produces the highest loss of SR. In this group, each additional percent of defect size leads to an average loss of SR of 1.3%. On the other hand, a low initial concavity results in a much lower loss of SR with a mean value of 0.6%.

**Table 2:** Results of linear models for three groups of initial concavity gradient

Initial concavity gradient	Initial SR			Loss of SR with increasing defect size			Model determination
	Mean	SE	95% CI	Mean	SE	95% CI	
Low, < 25%	29.7	1.6	[26.4, 33.0]	0.6	0.05	[0.7, 0.5]	*R*^2^ = 0.83
Medium, 25–35%	38.3	2.0	[34.3, 42.3]	0.8	0.04	[0.9, 0.7]	*R*^2^ = 0.93
High, > 35%	53.1	2.2	[48.6, 57.6]	1.3	0.06	[1.4, 1.1]	*R*^2^ = 0.95

**Fig. 8:**
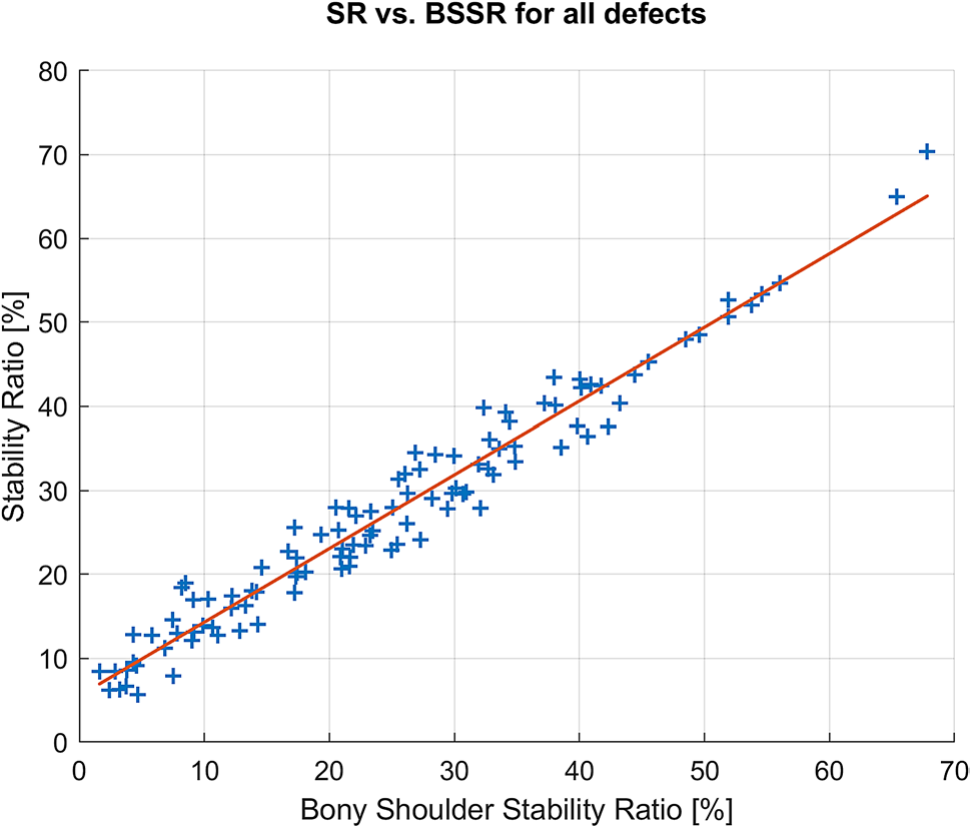
Linear model with BSSR as predictor for the SR; the BSSR is a highly correlated indicator for the measured SR independent of defect size

In a last step, the exclusive effect of the morphometrical-based BSSR on the measured SR was analysed with a linear model presented in Fig. 8. This linear approximation was determined independent of the defect size and resulted in a high determination coefficient of *R*^2^ = 0.98 and a low MSE of 3.4%. The linear coefficient was 0.9 ± 0.01 (95% CI [0.85, 0.91]) and differed significantly from zero (*p* < 0.001).

## Discussion

Three main findings can be summarised from the results of this study: (1) the SR is significantly dependent on glenoid concavity, whereas the defect size has a minor influence. (2) The loss of SR with each percentage of defect size is significantly dependent on the initial concavity. Therefore, a certain defect size can have different impacts on the stability. (3) For an estimation of the SR independent of defect size, the BSSR proved to be a reliable and most precise predictor.

Over the last several years, many studies have attempted to establish the most precise critical threshold of the defect size as a decision criterion for surgical treatment of shoulder instabilities [5, 8, 11, 16, 22, 25, 26, 29, 31]. Until now, a defect width of 20–25% was a commonly applied critical threshold [6, 8, 16, 29]. However, Shaha et al. [24] reported significantly inferior clinical outcomes for defect widths greater than 13.5%, if only soft tissue stabilisation was performed. Shin et al. [25] reported a significant loss of glenohumeral stability at a defect size of 7.5% according to 2 mm glenoid width. In addition to a successive adjustment of the critical threshold, some studies based on CT scans and FEM simulations have recently indicated that the defect size alone is insufficient to assess the SR, a biomechanical estimate of glenohumeral stability. Moroder et al. concluded that the biomechanical effect of glenoid defects varies with interindividual constitutional glenoid shape differences. To achieve a better estimation of the remaining stability, they derived the bony shoulder stability ratio (BSSR) and provided new insights in the discussion about a critical threshold. However, to date, there has been no biomechanical study examining the influence of defect size and glenoid concavity, even though many studies focusing on glenohumeral instabilities refer to concavity compression as a main stabiliser in the mid-range of motion [6–8, 13–15].

In this experimental study, a crucial biomechanical influence of glenoid concavity on the SR was demonstrated, whereas the defect size measurement had minor explanatory power. The results reinforce the statement of Moroder et al. that the biomechanical effect of a certain defect size varies with constitutional glenoid shape differences [19]. Furthermore, the results explain why there is no consensus to date in the search for a single threshold and the defect size measurement that is well suited for all patients [28]. For this reason, a single critical threshold of the defect size seems inappropriate for decision-making in surgical treatment. In contrast, a clinical inclusion of concavity information may allow for a more personalised and optimised treatment plan.

The classification of specimens into three groups according to their initial concavity revealed that a concavity is associated with a higher loss of SR for a certain size of bony defect. Hence, a 20% bone defect can result in a total loss of SR of 25% for high initial concavity gradients, or in a total loss of SR of 11% for low initial concavity gradients. Furthermore, the SR of 3 specimens in the intact state was already lower than the residual SR of glenoids, which had a high concavity but were affected by a defect size of 20%. These relations indicate that without including concavity information, it is not possible to accurately estimate remaining stability when a surgeon is faced with a 20% bone loss. In the event of a large concavity, the bony structure can provide more stability despite a certain bony defect compared to a flat glenoid even in the intact state. Therefore, it is conceivable that arthroscopic soft tissue reconstruction may be adequate for stabilisation in bony defects of 20% if the glenoid has a distinct concavity. On the other hand, bony reconstruction may be preferred for much smaller defects if the glenoid shape is very flat. Since the stabilising role of the surrounding soft tissue remains unclear in this context, further investigation is needed. In addition, the correlation with clinical outcomes is needed to determine an appropriate critical concavity threshold below which bony reconstruction should be preferred.

In this study, the concavity gradient was established since neither the effective depth, nor the effective width can provide a separate estimate of the glenoid curvature [19]. However, the gradient of curvature is hard to determine in clinical practice. Therefore, the morphometrical-based BSSR was also evaluated [19, 20]. This mathematical approximation of the measured SR achieved the highest correlation and fewest errors independent of the defect size. The BSSR is computable from glenoid depth and the sphere radius of the humeral head. As both values can be measured in CT data, the BSSR represents a proper parameter for a clinical application in the future. However, it is important to note that the BSSR as defined by Moroder et al. refers only to the bony structure as captured in CT data. In this study, the osteochondral surfaces were examined in the calculation of the BSSR, and good coverage of the biomechanical properties was demonstrated. To assess the SR in clinical practice, the bony structure and the cartilage must be considered by a proper method to calculate the BSSR.

A major limitation of this study is that all soft tissues were dissected from the specimens. Under physiological conditions, glenohumeral stability is provided by both the surrounding soft tissues and the articular surface. It is known that the labrum contributes to a large extent to glenohumeral stability by increasing the effective glenoid depth [7, 13]. Nonetheless, it was decided to dissect the labrum, as its stabilising effect had been extensively investigated [7, 8, 10, 13–15, 22]. As in comparable studies focusing on isolated bony effects, influences of the labrum should be avoided [25, 30]. In addition to the labrum, capsuloligamentous and muscular restraints also contribute to stability, especially during extreme and complex movements. For this reason, the test was performed in a position where soft tissues are known to have a minor influence on the overall stability [13]. Furthermore, the aim of this study was to analyse the biomechanical influences of the bony glenoid structure and to compare the usual clinical treatment based on a defect size measurement with new findings on concavity. In a clinical situation with glenoid bone loss, surgical treatment is mainly based on defect size, with less focus on the soft tissues. However, as shown in this study, concavity rather than defect size should be used to assess bony stabilisation. Thus, even without considering the soft tissues, the results are relevant for improvements in clinical decision-making in the treatment of glenoid bone loss.

Another limitation of this study is that the CP was cut off before testing. For a translation in the 3 o’clock direction, the CP usually has no resistive effect on the humeral head. However, the humeral head might be deflected inferiorly by a small coracohumeral interval during anterior translation. Pilot tests revealed that this effect would lead to unreliable and disturbed measurements. To focus on concavity, defect size and stability, the CP was resected, resulting in this trade-off. Nevertheless, the higher predictability of SR by concavity is expected to persist, even if a deflection might occur by the CP. Finally, the high age of specimens is another limitation. To reduce the effects of chondral and bony defects due to pre-existing conditions such as osteoarthritis, only the cadaveric glenoids were tested. Best-fit humeral implants were used as a standardised counterpart, as the spheres of glenoid and humeral head fit very well [9, 27]. With this approach, it was assumed that the results would apply also to younger patients who are physically active and who most commonly experience glenoid bone loss. However, it follows that only bony defects at the glenoid, and not at the humeral head, could be considered. The effects of a Hill–Sachs lesion or bipolar bone loss remain unexplored in terms of concavity.

## Conclusion

Glenoid concavity is a crucial factor for the SR, a biomechanical estimate of glenohumeral stability. Inclusion of concavity allows a more precise assessment of the SR than the defect size alone. The computable BSSR is a reliable and accurate biomechanical estimate of the measured SR independent of anterior defect size. In the future, this may allow for an improved and personalised treatment of shoulder instability in clinical daily routine.

## Supplementary Information

Below is the link to the electronic supplementary material.

Supplementary file1 (MP4 97103 kb)

## Abbreviations

BSSR: Bony shoulder stability ratio
CP: Coracoid process
CT: Computed tomography
FEM: Finite-element method
MSE: Mean squared error
SR: Stability ratio

## References

[1] Bergmann G, Graichen F, Bender A, Rohlmann A, Halder A, Beier A, Westerhoff P (2011). In vivo gleno-humeral joint loads during forward flexion and abduction. J Biomech.

[2] Bockmann B, Venjakob AJ, Reichwein F, Hagenacker M, Nebelung W (2017). Mapping of glenoid bone loss in recurrent anterior shoulder instability: is there a particular deficit pattern?. J Shoulder Elbow Surg.

[3] Burkhart SS, De Beer JF (2000). Traumatic glenohumeral bone defects and their relationship to failure of arthroscopic Bankart repairs. Arthroscopy.

[4] Di Giacomo G, Pugliese M, Lie DTT, Chou ACC, Chen J, Rosenberg N, Itoi E (2020). How to handle minor and major bone loss in the shoulder? Current concepts. J ISAKOS.

[5] Gottschalk LJ, Walia P, Patel RM, Kuklis M, Jones MH, Fening SD, Miniaci A (2016). Stability of the glenohumeral joint with combined humeral head and glenoid defects. Am J Sports Med.

[6] Greenstein AS, Chen RE, Knapp E, Brown AM, Roberts A, Awad HA, Voloshin I (2021). A biomechanical, cadaveric evaluation of single- versus double-row repair techniques on stability of bony bankart lesions. Am J Sports Med.

[7] Halder AM, Kuhl SG, Zobitz ME, Larson D, An KN (2001). Effects of the glenoid labrum and glenohumeral abduction on stability of the shoulder joint through concavity-compression. J Bone Joint Surg.

[8] Itoi E, Lee S-B, Berglund LJ, BergeE LL, An K-N (2000). The effect of a glenoid defect on anteroinferior stability of the shoulder after Bankart repair: a cadaveric study. J Bone Joint Surg.

[9] Kelkar R, Wang VM, Flatow EL, Newton PM, Ateshian GA, Bigliani LU, Pawluk RJ, Mow VC (2001). Glenohumeral mechanics: a study of articular geometry, contact, and kinematics. J Shoulder Elbow Surg.

[10] Klemt C, Nolte D, Grigoriadis G, Di Federico E, Reilly P, Bull AMJ (2017). The contribution of the glenoid labrum to glenohumeral stability under physiological joint loading using finite element analysis. Comput Methods Biomech Biomed Engin.

[11] Klemt C, Toderita D, Nolte D, Di Federico E, Reilly P, Bull AMJ (2019). The critical size of a defect in the glenoid causing anterior instability of the shoulder after a Bankart repair, under physiological joint loading. Bone Joint J.

[12] Lacheta L, Herbst E, Voss A, Braun S, Jungmann P, Millett PJ, Imhoff A, Martetschläger F (2019). Insufficient consensus regarding circle size and bone loss width using the ratio—“best fit circle”—method even with three-dimensional computed tomography. Knee Surg Sports TraumatolArthrosc.

[13] Lazarus MD, Sidles JA, Harryman DT, Matsen FA (1996). Effect of a chondral-labral defect on glenoid concavity and glenohumeral stability. A cadaveric model. J Bone Joint Surg.

[14] Lippitt S, Matsen F (1993). Mechanisms of glenohumeral joint stability. ClinOrthopRelat Res.

[15] Lippitt SB, Vanderhooft JE, Harris SL, Sidles JA, Harryman DT, Matsen FA (1993). Glenohumeral stability from concavity-compression: a quantitative analysis. J Shoulder ElbSurg.

[16] Lo IKY, Parten PM, Burkhart SS (2004). The inverted pear glenoid: an indicator of significant glenoid bone loss. Arthroscopy.

[17] Ludewig PM, Phadke V, Braman JP, Hassett DR, Cieminski CJ, LaPrade RF (2009). Motion of the shoulder complex during multiplanar humeral elevation. J Bone Joint Surg.

[18] Moroder P (2020). Editorial commentary: glenoid bone loss measurements in shoulder instability—precise but not accurate. Arthroscopy.

[19] Moroder P, Damm P, Wierer G, Böhm E, Minkus M, Plachel F, Märdian S, Scheibel M, Khatamirad M (2019). Challenging the current concept of critical glenoid bone loss in shoulder instability: does the size measurement really tell it all?. Am J Sports Med.

[20] Moroder P, Ernstbrunner L, Pomwenger W, Oberhauser F, Hitzl W, Tauber M, Resch H, Moroder R (2015). Anterior shoulder instability is associated with an underlying deficiency of the bony glenoid concavity. Arthroscopy.

[21] Moroder P, Haniel F, Quirchmayr M, Schulz E, Eppel M, Matis N, Auffarth A, Resch H (2016). Effect of glenoid concavity loss on shoulder stability- a case report in a professional wrestler. BMC MusculoskeletDisord.

[22] Nacca C, Gil JA, Badida R, Crisco JJ, Owens BD (2018). Critical glenoid bone loss in posterior shoulder instability. Am J Sports Med.

[23] Saito H, Itoi E, Sugaya H, Minagawa H, Yamamoto N, Tuoheti Y (2005). Location of the glenoid defect in shoulders with recurrent anterior dislocation. Am J Sports Med.

[24] Shaha JS, Cook JB, Song DJ, Rowles DJ, Bottoni CR, Shaha SH, Tokish JM (2015). Redefining “critical” bone loss in shoulder instability. Am J Sports Med.

[25] Shin S-J, Ko YW, Scott J, McGarry MH, Lee TQ (2016). The effect of defect orientation and size on glenohumeral instability: a biomechanical analysis. Knee Surg Sports TraumatolArthrosc.

[26] Shin S-J, Koh YW, Bui C, Jeong WK, Akeda M, Cho NS, McGarry MH, Lee TQ (2016). What is the critical value of glenoid bone loss at which soft tissue Bankart repair does not restore glenohumeral translation, restricts range of motion, and leads to abnormal humeral head position?. Am J Sports Med.

[27] Soslowsky LJ, Flatow EL, Bigliani LU, Mow VC (1992). Articular geometry of the glenohumeral joint. ClinOrthopRelat Res.

[28] Verweij LPE, Schuit AA, Kerkhoffs GMMJ, Blankevoort L, van den Bekerom MPJ, van Deurzen DFP (2020). Accuracy of currently available methods in quantifying anterior glenoid bone loss: controversy regarding gold standard—a systematic review. Arthroscopy.

[29] Yamamoto N, Itoi E, Abe H, Kikuchi K, Seki N, Minagawa H, Tuoheti Y (2009). Effect of an anterior glenoid defect on anterior shoulder stability. Am J Sports Med.

[30] Yamamoto N, Itoi E, Abe H, Kikuchi K, Seki N, Minagawa H, Tuoheti Y (2009). Effect of an anterior glenoid defect on anterior shoulder stability: a cadaveric study. Am J Sports Med.

[31] Yamamoto N, Muraki T, Sperling JW, Steinmann SP, Cofield RH, Itoi E, An K-N (2010). Stabilizing mechanism in bone-grafting of a large glenoid defect. J Bone Joint Surg.

